# Assessment of the GHG Reduction Potential from Energy Crops Using a Combined LCA and Biogeochemical Process Models: A Review

**DOI:** 10.1155/2014/537826

**Published:** 2014-06-17

**Authors:** Dong Jiang, Mengmeng Hao, Jingying Fu, Qiao Wang, Yaohuan Huang, Xinyu Fu

**Affiliations:** ^1^Institute of Geographical Sciences and Natural Resources Research, Chinese Academy of Sciences, 11A Datun Road, Chaoyang District, Beijing 100101, China; ^2^University of Chinese Academy of Sciences, Beijing 100049, China; ^3^Satellite Environmental Application Center, Ministry of Environmental Protection, Beijing 100094, China

## Abstract

The main purpose for developing biofuel is to reduce GHG (greenhouse gas) emissions, but the comprehensive environmental impact of such fuels is not clear. Life cycle analysis (LCA), as a complete comprehensive analysis method, has been widely used in bioenergy assessment studies. Great efforts have been directed toward establishing an efficient method for comprehensively estimating the greenhouse gas (GHG) emission reduction potential from the large-scale cultivation of energy plants by combining LCA with ecosystem/biogeochemical process models. LCA presents a general framework for evaluating the energy consumption and GHG emission from energy crop planting, yield acquisition, production, product use, and postprocessing. Meanwhile, ecosystem/biogeochemical process models are adopted to simulate the fluxes and storage of energy, water, carbon, and nitrogen in the soil-plant (energy crops) soil continuum. Although clear progress has been made in recent years, some problems still exist in current studies and should be addressed. This paper reviews the state-of-the-art method for estimating GHG emission reduction through developing energy crops and introduces in detail a new approach for assessing GHG emission reduction by combining LCA with biogeochemical process models. The main achievements of this study along with the problems in current studies are described and discussed.

## 1. Introduction

The increasing consumption of fossil fuel and current ecological environmental problems are global challenges. Plant-based bioenergy liquid fuel (including biofuel ethanol and biodiesel) is an effective way to relieve the energy crisis and also protect the environment due to its advantages of cleanness, safety, and reproducibility [1, 2]. After nearly 10 years, the worldwide production of liquid fuel is developing very rapidly, increasing from 0.96 billion in 2001 to 21.4 billion in 2011. The European Union, the USA, and Brazil are the main forces in the development of the biomass energy industry [3]. Although the development of the global biofuel industry has shown a great trend driven by the energy requirement and related policies, there are still many challenges in large-scale production. The main raw material of liquid biofuel production is currently cultivated crops. Soybean and corn are widely used in the USA and rapeseed and soybean are used in the European Union for biodiesel development. In Brazil, sugarcane is used for ethanol development [4]. Relatively accurate conclusions regarding productivity and environmental benefits may be drawn based on years of cultivated experience. The production of bioethanol and biodiesel by different energy plants and process techniques can reduce greenhouse gas (GHG) emissions by 12–125% compared with traditional fossil fuels [5–7]. Adler et al. used the DAYCENT biogeochemistry model to assess the soil GHG fluxes and biomass yields for corn, soybean, alfalfa, hybrid poplar, reed canarygrass, and switchgrass as bioenergy crops in Pennsylvania, USA. The results showed that all cropping systems considered provided net GHG sinks. The net GHG emissions of switchgrass, reed canarygrass, corn-soybean rotation, corn-soybean-alfalfa rotation, and hybrid poplar were reduced by −114%, −84%, −38%, −41%, and −117%, respectively [6]. Large-scale production of biodiesel in the UK was found to save 26% of GWP [8]. However, Jatropha and other noncrop energy plants have not been used long enough to generate sufficient data. The key problem of noncrop energy plants scale development is how to scientifically estimate the potential of GHG emission reduction [9]. If this problem can be solved, the development of biological liquid fuels can be more accurately evaluated and more reasonably planned.

The main purpose for the development of biological liquid fuel is to reduce GHG emissions, but great uncertainty remains regarding its comprehensive environmental impact. Some researchers believe that the patterns of land use change will affect the GHG emissions. These researchers believe that biological liquid fuel development would have a negative impact on the environment if the GHG emissions caused by the land use pattern changes were under consideration [10, 11]. However, according to the latest survey of the American Department of Energy, certain assumptions in the studies above have obvious problems. They assumed that 30 billion gallons of ethanol would be produced from corn annually until 2015, but only 1.5 billion gallons were planned to be produced according to the Energy Independence and Security Act [12]. They also assumed that massive deforestation would occur during the development of biomass energy, but most of the forests were excluded in the planning. Therefore, the assumption of a large amount of cultivated land being occupied is not correct because the biomass energy is developed based on the sparse forest land, sparse shrub land, sparse grassland, shoal/bottomland, and bare land rather than the cultivated land [13].

Regarding the net energy balance problems during production, ethanol from corn yields 25% more energy than the energy invested in its production, whereas biodiesel from soybeans yields 93% more [5]. Switchgrass produces 540% more bioethanol than nonrenewable energy consumed [12], which shows a great advantage of the second generation of biological liquid fuel. Some controversy also exists as to whether the development of bioliquid fuel will reduce GHG emissions. Some studies have indicated that GHG emissions can be reduced by 12–125% with bioliquid fuel production compared to traditional fossil fuels [12]. Bioethanol production from corn can reduce GHG emissions by 13%. The second generation biofuel can reduce more GHG emissions along with the development of process techniques [14]. Bioethanol production from switchgrass instead of fossil fuels can reduce GHG emissions by 94% [15]. Sasaki et al. [16] developed biomass change and harvest models to estimate the woody biomass availability in forests under the current management regime. The total annual production of woody biomass is 563.4 million tons (11.3 EJ) over the same period between 1990 and 2020. The total energy consumption in Southeast Asia was estimated at 6.4 EJ in 1990 and 15.7 EJ in 2006, increasing approximately by 9.0% yr^−1^. Energy from wood fuels in Southeast Asia (excluding Singapore and Brunei) was estimated at 2.4 EJ in 1993 or approximately 33.1% of the total energy consumption in that year. Energy from wood fuels in this region increased by approximately 2.5% yr^−1^ on average between 1992 and 1995 [17, 18]. Therefore, without effective policies to reduce deforestation and forest degradation, an energy shortage is likely to occur in Southeast Asia. The carbon emission reductions associated with using woody biomass instead of fossil fuels to generate energy are estimated at 281.7 TgC yr^−1^for replacing coal, 225.3 TgC yr^−1^ for replacing petroleum products, and 169.0 TgC yr^−1^ for replacing natural gas throughout the modeling period using carbon coefficients of 25 kgC GJ^−1^ for coal, 20 kgC GJ^−1^ for petroleum products, and 15 kgC GJ GJ^−1^ for natural gas [16].

Some controversy remains about the effects of biological liquid fuel development on the economy, society, and environment. Therefore, many countries have begun to reevaluate their future biofuel development strategies, exploring strategies that have smaller negative effects on the economy, society, and environment. For example, the European Union decided to postpone the implementation of their goal of replacing 10% of their transportation energy with biological liquid fuel by 2020, and the United States government claimed to assess and monitor the sustainability of biological liquid fuel development [19]. China's biofuel industry is also witnessing rapid development. However, the development of the biodiesel industry is still faced with many uncertainties, among which the accurate estimation of the potentiality of raw material supply, net energy production, and GHG emission reduction is the most crucial issue.

The main objectives of this study are the following: (1) to review the state-of-the-art method for assessing the GHG emission reduction by developing energy crops and (2) to introduce a new approach for assessing the GHG emission reduction by combining life cycle analysis (LCA) with biogeochemical process models. This paper focuses on estimating the GHG reduction of noncrop energy plants, especially in the stages of growing, managing, and harvesting. In addition, the GHG caused by direct land-use changes were considered, while the GHG caused by indirect land-use change were beyond the scope of this paper.

## 2. Life Cycle Analysis

To become a substitute for fossil fuels, bioliquid fuel should be able to provide net energy, bring environmental and economic benefits, and not reduce the food supply during mass production [6]. LCA is used to evaluate the energy consumption of a product or system throughout its life cycle, including raw material acquisition, production, product use, and postprocessing [3]. In recent years, LCA has been widely used as a complete comprehensive analysis method in bioenergy assessment studies. By comparison with fossil fuels, the consumption across the whole life cycle of biofuels, GHG emission, and primary energy usage can be reduced. Xing et al. [20] calculated and evaluated the land use and water and energy consumption of three feedstocks, namely, rape seed oil,* Jatropha curcas *L. oil, and waste oil, using LCA that considered planting, harvesting, transportation, pretreatment, biodiesel production, distribution, and consumption. Hu et al. established a life cycle energy consumption and emission assessment model for soybean, rape seed, Cornus wilsoniana wanaer (CWW), and* Jatropha curcas* L. as bases for biodiesel [21]. Wang and Lu analyzed the life cycle energy consumption and pollutant emissions during biodiesel production from* Jatropha curcas* [22]. The costs, energy consumption, and environmental impact of a bioethanol life cycle that used wheat, corn, and sweet potato as raw materials were analyzed by Zhang [23]. Dai et al. [24] evaluated the energy efficiency of the cassava fuel ethanol life cycle in the Guangxi province, China. Nguyen et al. [25] assessed the energy balance and GHG emissions of the cassava fuel ethanol life cycle in Thailand. Sobrino et al. [1] compared energy consumption of bioliquid fuels with fossil fuels throughout the life cycle and found a lower consumption of primary energy and a CO_2_ emission reduction after bioliquid fuel replaced certain fossil fuels. Razon and Tan [26] analyzed the net energy gain of bioliquid fuel and biogas using algae. Finally, Lu et al. [27] and Li et al. [28] established the energy and GHG reduction potential of* Pistacia chinensis*.

Most of the current literature is devoted to experimental or theoretical evaluations in estimating the GHG reduction effects of a certain energy plant for unit volume or mass using LCA. The mean value is used when applied on a district level [29]. The result is that the spatial differences in the GHG reduction potential resulting from the spatial heterogeneity of climate, soil, and terrain features cannot be determined. Hence, the GHG reduction potential is difficult to evaluate on a regional scale.

Addressing this problem, some studies proposed introducing spatial data and spatial analysis methods that couple LCA with GIS to evaluate the GHG reduction potential. A multifactor analysis method based on geographic information system (GIS) techniques was adopted to identify marginal lands for bioenergy development. Marginal lands with potential for planting energy plants were identified for each 1 km × 1 km grid across China. The net energy and emission reduction efficiency of biological liquid fuel were identified at each grid and the total GHG emission reduction was then obtained by accumulating the grids [30]. GIS techniques and multifactor comprehensive analysis methods are applied to calculate the potential for planting large-scale cassava in Southwest China. Then, the life cycle net energy and GHG emission reduction capacities of cassava on marginal land with different suitability degrees were calculated based on the expanded life cycle model for cassava ethanol fuel. The results indicate that adopting spatial data (such as the climate, soil, and terrain conditions) as well as a spatial analysis model provides a preliminary solution to solve the GHG reduction evaluation problem on a regional scale. The more reasonable results for GHG reduction potential were estimated at relatively fine geographical scale [31]. Dresen and Jandewerth integrated spatial analyses into LCA-calculated GHG emissions with GIS systems. Using the example of the energetic utilization of biomass via conditioned biogas, the authors presented a GIS-based calculation tool that combines geodata on biomass potentials, infrastructure, land use, cost, and technology with analysis tools for the planning of biogas plants to identify the most efficient plant locations and to calculate the emission balances, biomass streams, and costs in the lower Rhine region and the Altmark region in Germany. The results of the GHG balances were presented. The balances of the individual sites, the regional balances, and their temporal development can be calculated in GIS using LCA methods. GIS tools not only allow the assessment of individual plants but also allow the determination of the GHG reduction potential, the biogas potential, and the necessary investment costs for entire regions. Thus, exploiting regional biogas potentials in a way that is sustainable and climate-friendly becomes simple [32]. Environmental integration, such as GIS and LCA, provides a methodology capable of providing enough information and results to determine an energy crop implementation strategy for reducing the energy consumption and CO_2_ eq. emissions. The methodology was applied and verified in a study area in Catalonia (southern Europe). The results showed that a high impact reduction in GHG could be achieved annually (annual reduction of 1,954,904 Mg of CO_2_ eq.) [33]. However, some obvious problems remain in the current research. The same parameters were used in the “GHG reduction efficiency” model without considering natural or social conditions. Meanwhile, the total GHG emission reduction potential is not exactly equal to the sum of the grid values, as mutual influences and interactions exist between each grid [29].

## 3. Model

In recent years, many methods, including LCA, have been widely used in bioenergy assessment studies. However, previous studies typically only calculated the unit mass or unit area of a biofuel life cycle based on a laboratory dataset, that is, the “GHG emission reduction efficiency.” Regional total GHG emission reduction potentials were simply considered as “efficiency times total yield.” The spatiotemporal variation of environmental factors, such as solar radiation, temperature, soil, and water, was not well described in previous studies. To solve this problem, various models have been adopted for estimating the GHG emission of energy plants. IPCC Tier 1 provided a very practical method for calculating GHG emissions. Ecosystem process models and land surface models have also been used. According to the latest progress in this field, the process-based biogeochemical models were introduced into the framework of LCA to quantitatively calculate the C, N, and GHG emissions during the growth of energy plants and obtain their spatial distribution as well.

### 3.1. Three-Tier Approaches of IPCC

De Klein et al. (New Zealand) used a three-tier approach to estimate the nitrous oxide (N_2_O) emissions from managed soils, including indirect N_2_O emissions from the additions of N to land due to deposition and leaching and emissions of carbon dioxide (CO_2_) following the additions of liming materials and urea-containing fertilizer. In the most basic form, direct N_2_O emissions from managed soils are estimated using Tier 1 methods (*(A.1)*; see the appendix). If more detailed emission factors and corresponding activity data are available to a country than are presented in Tier 1, then Tier 2 can be undertaken. Tier 3 methods are modeling or measurement approaches that can relate the soil and environmental variables responsible for N_2_O emissions to the sizes of those emissions [34]. Tier 1 methods were most widely used because of the data acquisition convenience. Ruesch and Gibbs created a new global map using the IPCC Tier 1 method of biomass carbon stored in above- and below-ground living vegetation. However, the methods they employed are not directly linked to ground-based measures of carbon stocks and have not been validated with field data [35]. At the national level, the intergovernmental panel on climate change (IPCC) has produced a set of guidelines for estimating the GHG inventories at different tiers of quality, ranging from Tier 1 up to Tier 3. The biome averages used in the Tier 1 approach to estimate forest carbon stocks are freely and immediately available and currently provide the only source of globally consistent forest carbon information; however, there are uncertainties caused by natural disturbance, topography, microclimate, and soil type. Additionally, the estimates may be too high or too low for some locations. A study suggested that the default values used in this approach underestimate the carbon stocks for ecosystems, such as temperate moist forests [36–39]. In addition to the weaknesses above, the IPCC guidelines provide the default values of regular crops without the default values of most specific energy plants, such as sugarcane, Miscanthus and Cassava.

### 3.2. Ecosystem Process Model and Land Surface Model

Wu et al. used a modified version of the soil and water assessment tool (SWAT) as a basic tool to simulate a series of biofuel production scenarios involving crop rotation and land cover changes in the James River Basin of the Midwestern United States. The grasslands could be classified based on the simulations in terms of biomass productivity and nitrogen loads. The group further derived the relationship of biomass production targets and the resulting nitrogen loads, and they projected the annual average water yield NO_3_-N load and soil NO_3_-N concentration during the 18-year simulation period (1991–2008) [40]. PnET (photosynthetic/evapotranspiration model) is a nested series of models of carbon, water, and nitrogen dynamics for forest ecosystems. The models were developed and validated in the Northeastern USA at both the site and the grid level (to 1 km resolution) by Aber et al. [41]. To contribute toward more reliable estimates of the N_2_O source strength of tropical rainforest ecosystems on a regional scale, Kiese et al. modified a process-oriented biogeochemical model, PnET-N-DNDC, and parameterized it to simulate C and N turnover and the production of associated N_2_O emissions in and from tropical rainforest ecosystems. The daily simulated N_2_O emissions based on site data were in good agreement (model efficiencies up to 0.83) with field observations in the wet tropics of Australia and Costa Rica [42]. A simulation model, Wetland-DNDC, for C dynamics and methane (CH_4_) emissions in wetland ecosystems was reported; the model's main structure was adopted from PnET-N-DNDC. The model has been validated against various observations from three wetland sites in Northern America. The validation results agree with the field measurement data [43]. Predictions using PnET-II at the stand or community level indicated that the lumped parameter approach worked well at both large (i.e., multiple community types) and small (within community types) spatial scales [44, 45]. However, this type of approach will provide inaccurate parameter estimates without the right “mix” of species to offset over- and underestimates because the mixture of species resulted in a compensating error  [46].

### 3.3. Biogeochemical Process Models

DAYCENT is a daily time series biogeochemical model used in agroecosystems to simulate the fluxes of carbon and nitrogen in the atmosphere, vegetation, and soil [47, 48]. The model is a version of the CENTURY biogeochemical model using a daily scale. The DAYCENT land surface submodel simulated the soil water and soil temperature dynamics well for a variety of sites ranging from dry grassland, wet managed grassland, and wet crop land systems. The simulated results were compared with observed snow cover data, weekly 0–10 cm soil water data, daily AET data, and soil temperature data. The *r* ^2^ values from the observed versus simulated results were between 0.58 and 0.96 [49]. The ability of DAYCENT to simulate NPP, soil organic carbon, N_2_O emissions, and NO_3_ leaching has been tested with data from various native and managed systems [50–52]. The DAYCENT biogeochemical model was used to represent GHG emissions more realistically for nonrice major crop types (corn, wheat, and soybean). The results indicate a significant potential to the reduce GHG emissions from cropped soils and to increase yields. Using nitrification inhibitors and split fertilizer applications both led to increased (~6%) crop yields, but the inhibitor led to a larger reduction in N losses (~10%). No-till cultivation, which led to C storage, combined with nitrification inhibitors resulted in reduced GHG emissions of ~50% and increased crop yields of ~7%  [53]. DAYCENT, used in this study, is likely to be an improvement over the IPCC method that estimates N_2_O emissions based solely on N inputs and does not account for weather and soil class. However, the dataset used during the simulation was mapped to an extremely coarse resolution at 1.9olutio, and the nonspatial data (e.g., rates and dates of fertilizer applications) were assumed to be identical within crop types across regions. Lee et al. calibrated and validated DAYCENT and predicted the biomass yield potential of switchgrass across the Central Valley of California. Six common cultivars were calibrated using published data across the USA and validated with data generated from four field trials in California (2007–2009). After calibration and validation, the model explained 66–90% of observed yield variation in 2007–2009. The model (2.0–9.9 Mg ha^−1^ yr^−1^) agreed well with the observed yield variance (1.3–12.2 Mg ha^−1^ yr^−1^) in the establishment year. The Alamo and Kanlow cultivars were estimated to have biomass production potential within the Central Valley of California under the selected management practices. The biomass management options of switchgrass were suggested to differ depending on the temperature and on the yields of the different ecotypes [54].

RothC-26.3 was originally developed and parameterized to model the organic C turnover in arable topsoils from the Rothamsted long term field experiments, hence the name. The model uses a monthly time step to calculate the total organic carbon (t ha^−1^), microbial biomass carbon (t ha^−1^), and D14C (from which the equivalent radiocarbon age of the soil can be calculated) on timescales from years to centuries [55–58]. The model has been evaluated for a range of climates and vegetation types (e.g., cropland, grassland, and forests) and has been previously used for prediction on both regional and global scales [59–64]. Hillier et al. have conducted a study for England and Wales, using the yield maps of four bioenergy crops, Miscanthus (*Miscanthus giganteus*), short rotation coppice (SRC) poplar (*Populus trichocarpa* Torr. & Gray* P. trichocarpa*, var. Trichobel), winter wheat, and oilseed rape, with RothC to simulate the soil C turnover over a 20-year period. The GHG emissions from soil are placed in context with the life cycle emissions and then quantify the potential fossil fuel C that could be displaced. The GHG balance is estimated for each of the 12 land use change transitions associated with replacing arable, grassland, or forest/seminatural land with each of the four bioenergy crops. Miscanthus and SRC are likely to have a mostly beneficial impact in reducing GHG emissions, while oilseed rape and winter wheat have either a net GHG cost or only a marginal benefit [65].

Biome-BGC version 4.1.2 was provided by Peter Thornton at the National Center for Atmospheric Research (NCAR, sponsored by the National Science Foundation) and by the Numerical Terradynamic Simulation group (NTSG) at the University of Montana. The model is a computer model that simulates the storage and fluxes of water, carbon, and nitrogen within the vegetation, litter, and soil components of a terrestrial ecosystem. Biome-BGC is primarily a research tool and many versions have been developed for particular purposes [66]. Biome-BGC was applied to simulate the behavior of three Mediterranean species (*Quercus ilex* L.,* Quercus cerris* L., and* Pinus pinaster* Ait.)  [67]. The model was also adapted to managed stands with long term observations of biomass production. The exercise includes a model analysis for 33 stands exemplifying typical forest management of beech, oak, pine, and spruce, that is, the four major tree species important to Central-European forestry [68]. In this area, Schmid et al. analyzed the carbon dynamics along an altitudinal gradient across the alpine treeline; the analysis provided insights into the sensitivity of simulated average carbon pools to the changes in environmental factors [69]. The Biome-BGC model was also applied in a forested area of Sweden. The current carbon balance of the forested area and its sensitivity to global change was simulated [70]. Eastaugh et al. applied the species-specific adaptation of the biogeochemical model Biome-BGC to Norway spruce across a range of Austrian climatic change zones using the Austrian National Forest Inventory. The relative influence of current climate change on forest growth was quantified. At the national scale, climate change was found to have negligible effect on Norway spruce productivity, due in part to opposing effects at the regional level [71]. Based on the Biome-BGC model, a modified net primary productivity (NPP) calculation is used to estimate the* Jatropha curcas *Linnaeus (JCL) yields. A zoning scheme that considers land cover status and potential yield levels was formulated and used to evaluate the potential area and production of future plantations at the global, regional, and national levels. The estimated potential area of JCL plantations is 59–1486 million hectares worldwide and the potential production is 56–3613 million ton dry seed y^−1^ [72]. The Biome-BGC outputs are useful for the following: (1) establishing the amount and distribution of C storage by plants; (2) predicting the behavior of different ecosystems in cases of CO_2_ concentration changes in the air; (3) exploring the controls of water stress and drought on plant carbon balances; (4) exploring the interannual variability of climate on growing season; and (5) furnishing important parameters useful to managing ecosystems, particularly forests [67].

The models most used for energy plant GHG simulations are listed in Table 1. It is worth noting that hundreds of different types of models have been used in the literature. Table 1 presents only select models that are relatively operable and widely applied.

## 4. Discussion and Conclusion

During the last decade, great effort has been directed at establishing an efficient method for comprehensively estimating the GHG emission reduction potential from large-scale cultivation of energy plants by combining LCA with ecosystem/biogeochemical process models. LCA presents a general framework for evaluating the energy consumption and GHG emission from energy crop plantation, yield acquisition, production, product use, and postprocessing. Meanwhile, ecosystem/biogeochemical process models are adopted to simulate the fluxes and storage of energy, water, carbon, and nitrogen in the soil-plant (energy crops) soil continuum. Although clear progress has been made in recent years, some problems remain in current studies and should be addressed.

1. Localization of key parameters: in some of the “GHG reduction efficiency” models, such as [27, 31] the key parameters are derived from the reference values of the American Oregon National Laboratory. The same parameters were used without considering local geographical and social conditions. Good examples of models that incorporate geographical and social conditions were presented by Qin et al. [73] and Gelfand et al. [74]. The terrestrial ecosystem model (TEM), a process-based global-scale ecosystem model, was used to estimate the C fluxes and pool sizes of switchgrass and Miscanthus in China. For each crop, TEM was calibrated against driving data and the rate limiting parameters for several biogeochemical processes were obtained from the parameterization [73]. Many more details regarding the parameterization of the process model were also presented in [74]. To achieve qualified and reliable results, the localization of key parameters and sensitivity analysis are very important and worth greater attention in further studies.
2. Acquisition of spatially explicit estimations: the total GHG emission reduction potential is not simply equal to the sum of the grid values, as in the Biome-BGC model. Mutual influences and interactions exist between each grid [29]. For example, the Biome-BGC team recently presented a new model, the regional hydrological and ecological simulation system (RHESSys), that combines the terrestrial ecosystem process model Biome-BGC with spatially explicit meteorological information and the TOPMODEL hydrologic routing model to make spatial and temporal predictions of carbon, water, and nitrogen dynamics over landscapes [75]. Xu et al. suggested developing a spatially explicit agent-based LCA analysis framework for improving the environmental sustainability of bioenergy systems [76]. Hence, spatially explicit process-based biogeochemical models are much important for deriving both the amount and the spatial distribution of the C, N, and GHG emissions during the growth of energy plants. Using these models, the GHG reduction efficiency of scale development of energy plants can be accurately evaluated.
3. Assessment of the effect of management system. The effect of management system has been neglected in many existing models. However, the environmental policy integrated climate (EPIC), provided by Blackland Research & Extension Center and USDA Grassland, Soil, and Water Laboratory, could predict the effects of management decisions on soil, water, nutrient, and pesticide movements [77]. Gelfand et al. implemented an EPIC-based spatially explicit integrative modeling framework to simulate the yields of perennial species grown on marginal lands across the ten-state study area in the US north-central region [74]. The international institute for applied systems analysis (IIASA) suggests that EPIC has accurately simulated the agricultural conditions and practices for hundreds of years into the past, providing an excellent basis for projecting future trends in global change [78]. Therefore, more attention should be paid to the management system or the practice of energy crop plantation in future studies.

## Figures and Tables

**Table 1:** Models mostly used for GHG simulation of energy plant.

Model	Study object	Study area	Author(s)
Tier 1	Vegetation	Global	Ruesch and Gibbs 2008 [35]
	*Eucalyptus regnans* forests	Australian	Keith et al. 2009 [36]

SWAT	Biofuel	James River Basin of the Midwestern United States	Wu et al. 2012 [40]

PnET	Forest ecosystems	Northeastern USA	Aber et al. 2005 [41]
	Tropical rainforest ecosystems	Wet tropics of Australia and Costa Rica	Kiese et al. 2005 [42]
	Wetland	Northern America	Zhang et al. 2002 [43]

DAYCENT	Dry grassland, wet managed grassland, and wet crop land systems	Minneapolis, Minnesota, USA	Parton et al. 1994 [49]
	Crops	USA	del Grosso et al. 2005 [50]
	Corn, wheat, and soybean	Worldwide	del Grosso et al. 2009 [53]
	Switchgrass	The Central Valley of California	Lee et al. 2012 [54]

RothC	Cropland	European Russia and the Ukraine	Smith et al. 2007 [59]
	Nonwaterlogged soils	Germany, England, the USA, the Czech Republic, and Australia	Coleman et al. 1997 [60]
	Miscanthus, poplar, winter wheat, and oilseed rape	England and Wales	Hillier et al. 2009 [65]

Biome-BGC	*Quercus ilex* L., *Quercus cerris* L., and *Pinus pinaster* Ait.	The Mediterranean area	Chiesi et al. 2007 [67]
	Beech, oak, pine, and spruce	Central-European forestry	Cienciala and Tatarinov 2006 [68]
	Forest	Central-European forestry	Schmid et al. 2006 [69]
	Forest	Sweden	Lagergren et al. 2006 [70]

Biome	Norway spruce	Austrian	Eastaugh et al. 2011 [71]
	*Jatropha curcas* Linnaeus	Global	Li et al. 2010 [72]

## Appendix

Consider

(A.1) N2ODirect-N=N2O-NN inputs+N2O-NOS +N2O-NPRPN2O-NN inputs=[[(FSN+FON+FCR+FSOM)•EF1] +[(FSN+FON+FCR+FSOM)FR+EF1FR]]N2O-NOS=[(FOS,CG,Temp•EF2CG,Temp) +(FOS,CG,Trop•EF2F,Temp,NP) +(FOS.F.Temp,NR•EF2F,Temp,NR) +(FOS,F,Temp,NP•EF2F,Temp,np) +(FOS,F,Trop•EF2F,Trop)]N2O-NPRP=[(FPRP,CPP•EF3PRP,CPP) +(FPRP,SO•EF3PRP,SO)],

where

N_2_O_Direct_-N = annual direct N_2_O-N emissions produced from managed soils, kg N_2_O-N yr^−1^,
N_2_O-N_Ninputs_ = annual direct N_2_O-N emissions from N inputs to managed soils, kg N_2_O-N yr^−1^,
N_2_O-N_OS_ = annual direct N_2_O-N emissions from managed organic soils, kg N_2_O-N yr^−1^,
N_2_O-N_PRP_ = annual direct N_2_O-N emissions from urine and dung inputs to grazed soils, kg N_2_O-N yr^−1^,
*F*_SN_ = annual amount of synthetic fertilizer N applied to soils, kg N yr^−1^,
*F*_ON_ = annual amount of animal manure, compost, sewage sludge, and other organic N additions applied to soils (note: if including sewage sludge, cross-check with waste sector to ensure that the N_2_O emissions are not double-counted from the N in sewage sludge), kg N yr^−1^,
*F*_CR_ = annual amount of N in crop residues (above-ground and below-ground), including N-fixing crops, and from forage/pasture renewal, returned to soils, kg N yr^−1^,
*F*_SOM_ = annual amount of N in mineral soils that is mineralized, in association with loss of soil C from soil organic matter as a result of changes to land use or management, kg N yr^−1^,
*F*_OS_ = annual area of managed/drained organic soils, ha (note: the subscripts CG, F, Temp, Trop, NR, and NP refer to cropland and grassland, forest land, temperate, tropical, nutrient rich, and nutrient poor, resp.),
*F*_PRP_ = annual amount of urine and dung N deposited by grazing animals on pasture, range, and paddock, kg N yr^−1^ (note: the subscripts CPP and SO refer to cattle, poultry, and pigs and sheep and other animals, resp.),
EF_1_ = emission factor for N_2_O emissions from N inputs, kg N_2_O-N (kg N input)^−1^,
EF_1FR_ is the emission factor for N_2_O emissions from N inputs to flooded rice, kg N_2_O-N (kg N input)^−1^,
EF_2_ = emission factor for N_2_O emissions from drained/managed organic soils, kg N_2_O-N ha^−1^ yr^−1^; (note: the subscripts CG, F, Temp, Trop, NR, and NP refer to cropland and grassland, forest land, temperate, tropical, nutrient fich, and nutrient poor, resp.),
EF_3PRP_ = emission factor for N_2_O emissions from urine and dung N deposited on pasture, range, and paddock by grazing animals, kg N_2_O-N (kg N input)^−1^ (note: the subscripts CPP and SO refer to cattle, poultry, and pigs, and sheep and other animals, resp.).
